# Pulmonary function and physical performance in adolescent e-cigarette users: a narrative review

**DOI:** 10.3389/fpubh.2025.1703712

**Published:** 2025-11-13

**Authors:** Chen Li, Tianyuan Guan

**Affiliations:** 1 Department of Exercise Science and Exercise Physiology, Kent State University, Kent, OH, United States; 2 College of Public Health, Kent State University, Kent, OH, United States

**Keywords:** e-cigarettes, adolescents, pulmonary function, exercise tolerance, physical performance, respiratory health

## Abstract

Adolescent e-cigarette use has risen sharply worldwide and poses emerging threats to respiratory health. As adolescence represents a critical window for lung growth and aerobic capacity, inhalation of toxicants during this period may disrupt pulmonary development and exercise performance. This narrative review synthesizes current evidence on the physiological and behavioral consequences of vaping in youth. Clinical and epidemiological studies indicate that adolescents who vape report higher rates of respiratory symptoms, such as chronic cough, wheezing, and shortness of breath, and may exhibit early declines in lung function and exercise tolerance. Experimental and mechanistic studies implicate nicotine, ultrafine particles, aldehydes, and flavoring agents in oxidative stress, airway inflammation, immune suppression, and impaired alveolarization. Dual users of e-cigarettes and combustible cigarettes appear at even greater risk, with lower cardiorespiratory fitness and muscular endurance compared with non-users. Despite these concerns, research is limited by cross-sectional designs, self-reported data, and a lack of longitudinal cohorts. Further high-quality studies are needed to determine the long-term impact of vaping on adolescent lung development and physical fitness. Preventive efforts in public health, education, and policy are critical to protect youth from the potential pulmonary hazards of e-cigarette use.

## 1 Introduction

The prevalence of e-cigarette use among adolescents has risen dramatically worldwide, often surpassing rates observed in adults and creating a major public health concern (1). In the United States, for example, current use among high school students surged from 1.5% in 2011 to 27.5% in 2019, prompting public health authorities to describe an “epidemic” of youth vaping (2). Similar upward trends have been reported globally, with many countries documenting double-digit prevalence in teenagers (3).

Adolescence is a critical developmental period for the lungs, during which lung volumes and functional capacity continue to increase until early adulthood (4). Exposure to inhaled toxicants during this window may hinder the attainment of maximal pulmonary function, with potential long-term consequences for respiratory health and physical performance (5–7). E-cigarettes generate an inhalable aerosol that contains nicotine along with multiple toxic chemicals and ultrafine particles capable of reaching deep into the lungs (8). In addition to nicotine’s systemic effects, constituents such as volatile organic compounds, heavy metals, and certain flavoring agents raise concerns for respiratory injury (9–11). These exposures highlight the potential for vaping to compromise adolescent lung health, underscoring the importance of further investigation.

Emerging evidence links adolescent vaping with measurable adverse effects on respiratory health and function. Population-based studies have found that youths who use e-cigarettes experience higher rates of chronic respiratory symptoms. For instance, a pooled analysis of US adolescent cohorts showed that teenagers vaping on >5 days per month had significantly higher odds of developing persistent bronchitic symptoms (such as chronic cough and phlegm) and shortness of breath compared to never-users (12). Similarly, epidemiologic data indicate an association between e-cigarette use and asthma in young people. One large survey of never-smokers reported that current e-cigarette users had about 39% higher odds of self-reported asthma than non-users, with a dose–response relationship (daily vapers showed the greatest risk increase) (13). More acute clinical reports have further underscored vaping’s potential for harm: in 2019, an outbreak of e-cigarette or vaping-associated acute lung injury (EVALI) in the US resulted in over 2,800 hospitalizations and 68 confirmed deaths, predominantly in adolescents and young adults (14). Taken together, current findings, from symptoms and airway reactivity to rare catastrophic injuries, implicate e-cigarette use as detrimental to adolescent pulmonary function and respiratory well-being.

Despite these concerning indications, much remains unknown about the long-term consequences of adolescent vaping on lung health. Because e-cigarettes are relatively new products, longitudinal studies tracking their respiratory impact are scarce; most research so far has been cross-sectional or limited to short-term observations (15). No definitive data yet exist on whether vaping during the teenage years leads to chronic impairment in lung growth or lasting deficits in adult pulmonary function. Many early studies also vary in design and outcome measures, making it challenging to draw firm conclusions. This significant gap in knowledge calls for further investigation: as experts have noted, understanding the full extent of e-cigarette effects on the developing lungs is critical for guiding public health policy and clinical recommendations (16). Given the rapid uptake of e-cigarettes among youth and the growing concerns regarding their health impact, a narrative review is warranted. This article synthesizes current evidence on adolescent e-cigarette use, with a focus on pulmonary function, exercise tolerance, and physical performance. It also highlights key gaps in the literature and outlines directions for future research to inform clinical practice and public health policy.

## 2 Methods

As this article is a narrative review, a systematic protocol such as PRISMA was not applied. Nevertheless, we followed a structured approach to ensure comprehensive coverage of relevant literature. Electronic searches were conducted in PubMed, Scopus, and Web of Science databases for studies published between 2010 and May 2025. The following keywords and combinations were used: “e-cigarette” OR “electronic cigarette” OR “vaping” AND “adolescent” OR “youth” OR “teenager” AND “pulmonary function” OR “lung function” OR “respiratory health” OR “exercise tolerance” OR “physical performance.”

To complement database searches, we reviewed the reference lists of relevant systematic reviews and policy documents. We included peer-reviewed original research articles, systematic reviews, and meta-analyses published in English. In addition, authoritative public health sources (e.g., Centers for Disease Control and Prevention, World Health Organization) were consulted to provide updated epidemiological data and policy context. Studies focusing exclusively on adult populations were excluded unless they provided mechanistic insights applicable to adolescents. Preference was given to clinical, epidemiological, and experimental studies reporting outcomes related to respiratory function, pulmonary development, or physical activity capacity.

The purpose of this search strategy was not to provide an exhaustive systematic synthesis, but rather to identify representative and recent studies that illuminate the potential impact of adolescent e-cigarette use on pulmonary health and exercise performance. Therefore, a PRISMA-style quantitative reporting of records identified, screened, and included was not applied, and no formal quality or risk-of-bias assessment was conducted, as the present work was designed as a qualitative narrative review rather than a systematic appraisal.

## 3 Epidemiology of e-cigarette use among adolescents

In the United States, e-cigarettes have been the most commonly used tobacco product among youth since around 2014, and their use rose sharply over the past decade, peaking in 2019 when 27.5% of high school students reported vaping (17). In that peak year, more than 5 million US middle- and high-school students were current e-cigarette users (with nearly 1 million vaping daily), a dramatic surge that reversed prior declines in overall youth tobacco use (18). Following this “epidemic” peak, adolescent vaping rates have declined but remain a major public health concern; for example, 14.1% of US high school students were current e-cigarette users in 2022, dropping to about 10% in 2023 (19). Even with recent declines, e-cigarettes continue to dominate youth tobacco use patterns in the US, far outpacing conventional cigarettes (20).

Globally, the prevalence of adolescent e-cigarette use has risen in tandem with the US trend (21). Recent estimates put the worldwide average of current vaping among youths at roughly 9%, with national figures ranging from under 2% in some countries to over 30% in others (22). In Europe, e-cigarette use among adolescents aged 13–15 years varies significantly, from less than 1% in some countries to over 20% in others, with several nations reporting that youth vaping now surpasses cigarette smoking in popularity (23). By contrast, many Asian countries have recorded lower youth vaping rates: surveys in Japan and South Korea found only about 3.5 and 10%, respectively, of adolescents had ever tried e-cigarettes, and a study in China observed just ~1% current use among middle-school students (24–26). Overall, despite regional differences, the emergence of e-cigarettes has become a global phenomenon among youth, with rapid uptake observed across North America, Europe, and other parts of the world.

Within adolescent populations, e-cigarette use exhibits notable demographic patterns. In recent US surveys, female high school students have shown slightly higher vaping prevalence than their male counterparts (e.g., approximately 10.5% vs. 8.4% in 2022) (27). Some data indicate that American Indian/Alaska Native youth have among the highest vaping rates, whereas Asian American youth have the lowest, though all groups have been affected by the rise of e-cigarettes (20). Socioeconomic status (SES) appears to play a role as well: studies generally find that adolescents from more socioeconomically disadvantaged backgrounds are more likely to vape than those from higher-SES families (28). These demographic trends suggest that the e-cigarette epidemic has not impacted all adolescent subgroups equally, and they underscore the importance of tailored public health strategies.

Multiple behavioral and environmental factors contribute to the popularity of e-cigarettes among adolescents. Research has shown that youth vaping is driven by a combination of targeted marketing, the availability of appealing flavored products, social influences, and adolescents’ misperceptions about risk (29, 30). E-cigarette advertising is pervasive, and the majority of US adolescents report frequent exposure to such promotions (31). This exposure has been consistently associated with increased likelihood of e-cigarette use among youth (32). Such pervasive marketing, combined with fruity and sweet e-liquid flavors that attract young tastes, has created an environment in which vaping is perceived as fashionable or less harmful by many adolescents. Addressing these behavioral and environmental drivers—through stricter marketing regulations, flavor restrictions, education about nicotine risks, and interventions in schools and on social media—is considered critical for curbing youth e-cigarette use (31).

## 4 Composition of aerosol and toxicology

**Figure 1:**
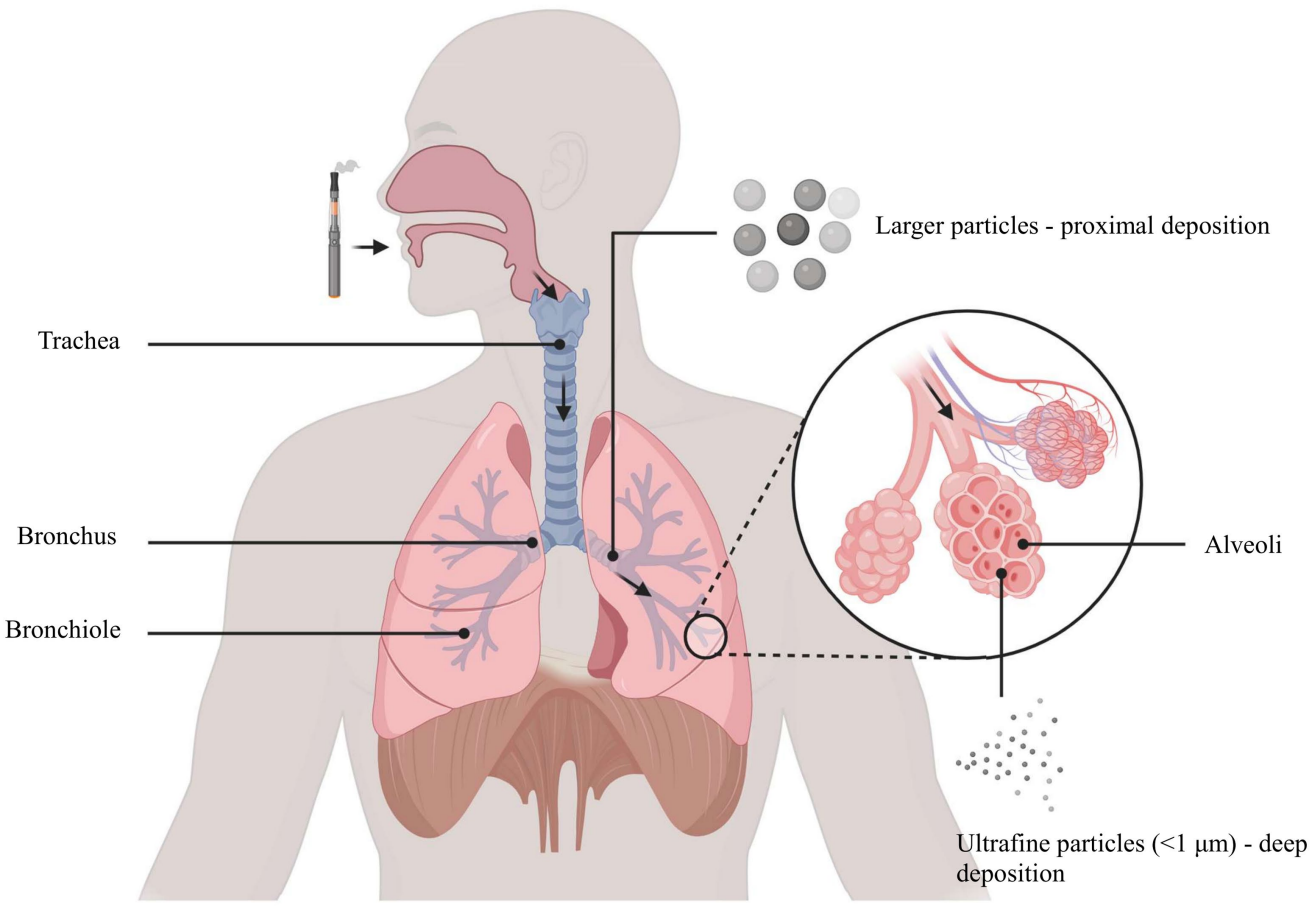
Ultrafine particles (<1 μm) penetrate into alveoli, while larger particles deposit primarily in proximal airways.

E-cigarette aerosol is a complex mixture generated by heating a liquid solution of propylene glycol (PG), vegetable glycerin (VG), nicotine, and flavoring agents (33). This process produces an inhalable vapor containing both the original ingredients and numerous toxic byproducts formed through thermal decomposition (34). A summary of key toxic constituents found in e-cigarette aerosol is provided in Table 1. Notably, carbonyl compounds such as formaldehyde, acetaldehyde, and acrolein are released when PG/VG solvents are heated, and these reactive aldehydes are well-recognized respiratory irritants and potential carcinogens (34). E-cigarette vapor analyses have also detected volatile organic compounds (VOCs; e.g., benzene) and heavy metals including lead, cadmium, and nickel in the aerosol (35, 36). Even though e-cigarette emissions generally contain fewer distinct chemicals than conventional cigarette smoke, they are by no means “safe,” as the inhaled particles are predominantly ultrafine in size and can deposit deeply in the lungs (37), as illustrated in Figure 1, where ultrafine particles travel through the airways and accumulate in the distal alveolar regions. Each 2-s puff of an e-cigarette delivers 6.25 × 10^10^ ultrafine particles into the airways, raising concerns about long-term particulate exposure in adolescent users (38).

**Table 1:** Summary of key chemical constituents and device-related factors in e-cigarette aerosol, their sources of formation, and reported respiratory health impacts.

Constituent/Factor	Source/formation	Respiratory impact	Representative references
Nicotine	E-liquids (nicotine salts, high-capacity pods)	Strong addiction potential, impaired lung and brain development	(39–41)
Carbonyls (formaldehyde, acetaldehyde, acrolein)	Thermal decomposition of PG/VG	Airway irritation, carcinogenicity, cytotoxicity	(34, 38)
Volatile organic compounds (benzene, etc.)	Solvent breakdown, heating	Systemic toxicity, respiratory irritation	(36)
Heavy metals (lead, cadmium, nickel, chromium)	Coil and device components	Chronic inflammation, fibrosis, cancer risk	(35, 49)
Ultrafine particles (<1 μm)	Aerosol droplets	Deep lung deposition, airway resistance, reduced lung function	(37)
Flavoring agents (vanillin, benzaldehyde, cinnamaldehyde)	E-liquid additives, vaporization reactions	Epithelial damage, immune suppression, cytotoxicity	(42–46)
Diacetyl	Sweet/dessert flavor additives	Bronchiolitis obliterans (“popcorn lung”), irreversible airway scarring	(11, 47)
Device characteristics (sub-ohm, disposables)	High power output, coil design	Increased yields of aldehydes, metals, VOCs	(38, 48)

Nicotine is typically present at high concentrations in adolescent-favored vaping products. Modern nicotine salt formulations in disposable and pod-style e-cigarettes often reach 5% or more, meaning a single high-capacity device can contain a total nicotine dose equivalent to an entire pack of cigarettes (39). This extraordinary nicotine content delivers powerful neuropharmacologic effects—young vapers can experience stronger addiction potential and withdrawal symptoms, reflecting nicotine’s well-documented capacity to impair adolescent brain and lung development and to induce rapid dependence (40, 41).

Beyond nicotine, the flavoring chemicals in e-liquids contribute significantly to aerosol composition and toxicity. Many flavor additives are compounds “Generally Recognized as Safe” (GRAS) for oral ingestion, yet their inhalation toxicity is largely uncharacterized (42). Emerging evidence shows that some flavor chemicals undergo chemical changes during vaporization or interact with carriers to form new toxicants. For example, aldehyde flavorings like vanillin, benzaldehyde, and cinnamaldehyde can react with PG/VG to form acetal compounds in the e-liquid, and 50–80% of these acetals transfer into the aerosol (43). Moreover, heat-driven chemical reactions yield a plethora of additional species: a recent AI-driven modeling study predicted that heating a panel of 180 common e-liquid flavor ingredients would generate 127 acutely toxic substances, 153 classified health hazards, and 225 irritant byproduct compounds in the vapor (44). This highlights the potential for flavored e-cigarettes to expose users to a vast array of chemical hazards that were not originally present in the liquid.

Many flavoring agents themselves are associated with direct toxicological effects on the respiratory system. Cinnamaldehyde (the compound imparting cinnamon flavor) is a prominent example: inhalation of cinnamon-flavored e-cigarette aerosol has been shown to damage airway epithelial cells and suppress pulmonary immune cell function, impairing alveolar macrophages’ ability to clear pathogens (45). Other sweet flavor chemicals have demonstrated similar harm; for instance, benzaldehyde (cherry flavor) and vanillin (vanilla) in e-cigarette vapor have been linked to reduced macrophage phagocytosis and cytotoxicity (46). Chronic inhalation of diacetyl is a known cause of bronchiolitis obliterans (the serious lung scarring disease nicknamed “popcorn lung”) in factory workers exposed to flavoring fumes, and this chemical has been detected in many sweet e-cigarette liquids (47). A 2021 risk assessment found that typical diacetyl exposure levels from e-cigarette use could result in hazard quotients up to 200–300 times above safe limits, strongly suggesting a significant risk for irreversible small-airway fibrosis (bronchiolitis obliterans) among teen and adult vapers of diacetyl-containing flavors (11). These findings underscore that flavor constituents, while benign in foods, may pose grave risks when inhaled over time.

Device characteristics and use patterns further modulate the chemical composition of e-cigarette aerosol. Power output and coil design have a profound influence on toxicant generation. Higher-temperature “sub-ohm” devices (using low-resistance coils and high wattage) produce dramatically greater yields of harmful chemicals compared to standard e-cigarettes. For example, experiments with a dessert-flavored e-liquid showed that vaping under sub-ohm conditions (<0.5 *Ω* coil) generated 7–15 μg of total carbonyl compounds per puff, including elevated formaldehyde, acetaldehyde, and acrolein, whereas the same liquid produced <2 μg/puff of carbonyls under more moderate power settings (38). Similarly, unregulated high-capacity disposable e-cigarettes (which lack electronic temperature control) have been reported to emit significantly higher levels of carbonyls and metals than regulated pod devices (48). Taken together, these findings indicate that device type, power level, and flavor composition collectively determine the chemical burden of e-cigarette aerosol, leading to wide variability in what an adolescent vaper might inhale.

Exposure to heavy metals originating from e-cigarette coils and components has been associated with adverse respiratory outcomes in adolescents. Inhaled metals such as nickel and chromium (commonly leached from heating coils) can deposit in lung tissue and have been associated with chronic airway inflammation, fibrosis, and elevated cancer risk (49). Finally, the ultrafine particulate matter in e-cigarette vapor may worsen respiratory health. E-cigarette aerosol particles (which are on the scale of <0.1 μm) penetrate deep into the bronchial and alveolar regions, where they can trigger airway irritation and inflammation even in the absence of active chemicals (37). The users of e-cigarettes have shown measurable increases in airway resistance and reductions in lung function metrics compared to non-users (50). Although the precise long-term clinical outcomes are still being researched, the toxicological profile of e-cigarette aerosol—rich in nicotine, oxidants, chemical irritants, and foreign particles—strongly implicates vaping in potential respiratory harm. Every component, from nicotine to flavor additives to metals, contributes to a mix of exposures that can adversely affect lung biology in adolescents, underscoring the need for caution and regulation in youth e-cigarette use.

## 5 Mechanisms of lung damage in adolescent E-cigarette users

Multiple mechanistic pathways underline the pulmonary damage observed in youth who vape, involving oxidative injury, inflammatory signaling, cellular toxicity, immune dysfunction, and interference with normal lung development (51). E-cigarette aerosol generates reactive oxygen species (ROS) in the airways that overwhelm antioxidant defenses and activate redox-sensitive inflammatory pathways (52). The resulting oxidative stress triggers the transcription factor NF-κB, leading to upregulated release of pro-inflammatory cytokines (e.g., IL-6, TNF-*α*) and chemokines that drive inflammation in the respiratory tract (52). In controlled animal models, even short-term inhalation of flavored e-cigarette vapor provoked acute lung inflammation (53). Repeated or chronic vaping sustains this state, which can contribute to persistent pulmonary inflammation, alveolar structural damage, and progressive impairment of lung function over time (54). Notably, the chemical constituents of e-cigarettes, including nicotine salts, aldehydes, and ultrafine particles, provoke bronchial epithelial irritation and oxidative stress, thereby amplifying epithelial injury and sustaining pulmonary inflammation (55).

E-cigarette exposure also inflicts DNA damage in lung cells and may impair the cells’ capacity for DNA repair. For instance, Lee et al. demonstrated that mice chronically exposed to e-cigarette aerosol accumulated elevated levels of DNA adducts in lung, bladder, and heart tissues, accompanied by a marked reduction in DNA repair enzyme activity, highlighting that both genetic integrity and repair capacity are compromised by such exposure (56). Repeated epithelial injury without proper repair can lead to maladaptive remodeling of the lung. Studies have noted that long-term vaping prompts structural changes in the lungs, including aberrant tissue remodeling and scarring in airways (54, 57). Fibroblasts may deposit excess extracellular matrix in injured areas, contributing to fibrotic changes that stiffen the lung and narrow the small airways (58). Thus, e-cigarettes can injure the respiratory epithelium both directly (through cytotoxic effects) and indirectly (through impaired healing and fibrotic repair), ultimately degrading the integrity and elasticity of the lung tissue.

Vaping also disrupts the lung’s innate immune defenses, which are critical during adolescence for protecting the developing respiratory system. Reidel et al. reported that chronic e-cigarette use was associated with elevated neutrophil elastase and matrix metalloproteinase-9 activity, accompanied by increased mucin concentrations, collectively reflecting impaired mucociliary clearance (59). Similarly, Clapp et al. demonstrated that the flavoring agent cinnamaldehyde markedly suppressed ciliary beat frequency and diminished phagocytic activity of immune cells, thereby directly compromising respiratory innate defense mechanisms (60). Furthermore, Madison et al. showed that e-cigarette exposure disrupted lipid homeostasis in alveolar macrophages, leading to the accumulation of lipid-laden macrophages with reduced capacity for pathogen clearance, independent of nicotine content (61). Taken together, these findings highlight that e-cigarettes compromise multiple components of airway innate immunity, thereby increasing susceptibility to respiratory dysfunction and infection.

Vaping during adolescence may stunt normal pulmonary development, as this developmental stage represents a critical window of lung growth characterized by ongoing alveolarization. Human imaging and stereological studies have demonstrated that alveolar formation continues throughout childhood and adolescence, underscoring the vulnerability of the developing lung to inhaled toxicants (62). Experimental evidence from neonatal and perinatal mouse models supports this concern. Exposure of neonatal mice to nicotine-containing e-cigarette aerosols impaired alveolarization, evidenced by enlarged mean linear intercepts and reduced cell proliferation (63). Similarly, maternal exposure to e-cigarette aerosols during pregnancy resulted in abnormal lung structure, emphysema-like changes, increased airway resistance, and dysregulated developmental gene expression in offspring (64). Translational studies in young adults also show functional impairments. In a controlled study of healthy young individuals, e-cigarette use was associated with an abnormal lung clearance index compared to non-users, indicating early impairment of small airway function detectable even prior to spirometric decline (65). Together, these findings highlight that adolescent vaping may interfere with normal pulmonary development by disrupting alveolar formation and inducing long-term structural and functional deficits.

In summary, the convergence of oxidative stress, epithelial injury, immune impairment, and developmental interference caused by e-cigarette use poses a serious threat to the respiratory health of young people. These mechanisms act in concert to weaken the structure and function of the adolescent lung, potentially leading to lasting deficits in pulmonary function and exercise capacity. Repeated vaping may lead to permanent lung damage and increase the likelihood of chronic lung diseases in this population. By undermining normal lung growth and defense, adolescent e-cigarette use may set the stage for respiratory limitations and vulnerabilities that persist into later life, underscoring the imperative to prevent vaping in youth and protect the lungs during their critical developmental years.

## 6 Pulmonary function and physical activity in adolescents: influence of e-cigarette use

### 6.1 Adolescent pulmonary function and exercise capacity

Adolescence is a critical period for lung development, during which pulmonary function metrics like forced expiratory volume in one second (FEV₁) and forced vital capacity (FVC) increase rapidly and reach their peak in early adulthood (66). The attainment of robust lung volumes and airflow during these years is important because it underpins aerobic capacity and endurance performance in youth. Higher baseline lung function allows for greater ventilation and oxygen uptake during exercise, which supports sustained physical activity. In fact, adolescents with superior cardiorespiratory fitness tend to have higher FEV₁ and FVC values, reflecting enhanced lung capacity that correlates with better exercise tolerance (67). This relationship is bidirectional: regular physical activity and sports participation can further improve pulmonary function. Engaging in high-intensity exercise training has been shown to induce significant gains in lung volumes during adolescence – for example, vigorous aerobic activity was associated with measurable increases in both FEV₁ and FVC in a recent adolescent cohort study (68).

Alongside spirometric lung volumes, the maximal oxygen uptake capacity (VO₂ max) also expands during the teenage years, driven by growth and hormonal changes of puberty (69). As youth progress through puberty, increases in heart and lung size, blood volume, and muscle mass all contribute to higher VO₂ max, thereby boosting overall exercise capacity. With appropriate training, adolescents can further augment their VO₂ max and endurance. Meta-analyses confirm that structured exercise programs lead to significant improvements in VO₂ max in children and adolescents, underscoring the plasticity of the developing cardiopulmonary system (70). Overall, achieving optimal lung function by late adolescence is not only a marker of respiratory health but also a facilitator of physical activity participation and sports performance. Conversely, suboptimal development of lung capacity in youth—due to sedentariness or other factors—can limit exercise endurance and even elevate risks of respiratory issues like exercise-induced bronchospasm or asthma (67).

### 6.2 E-cigarettes and youth lung function

**Figure 2:**
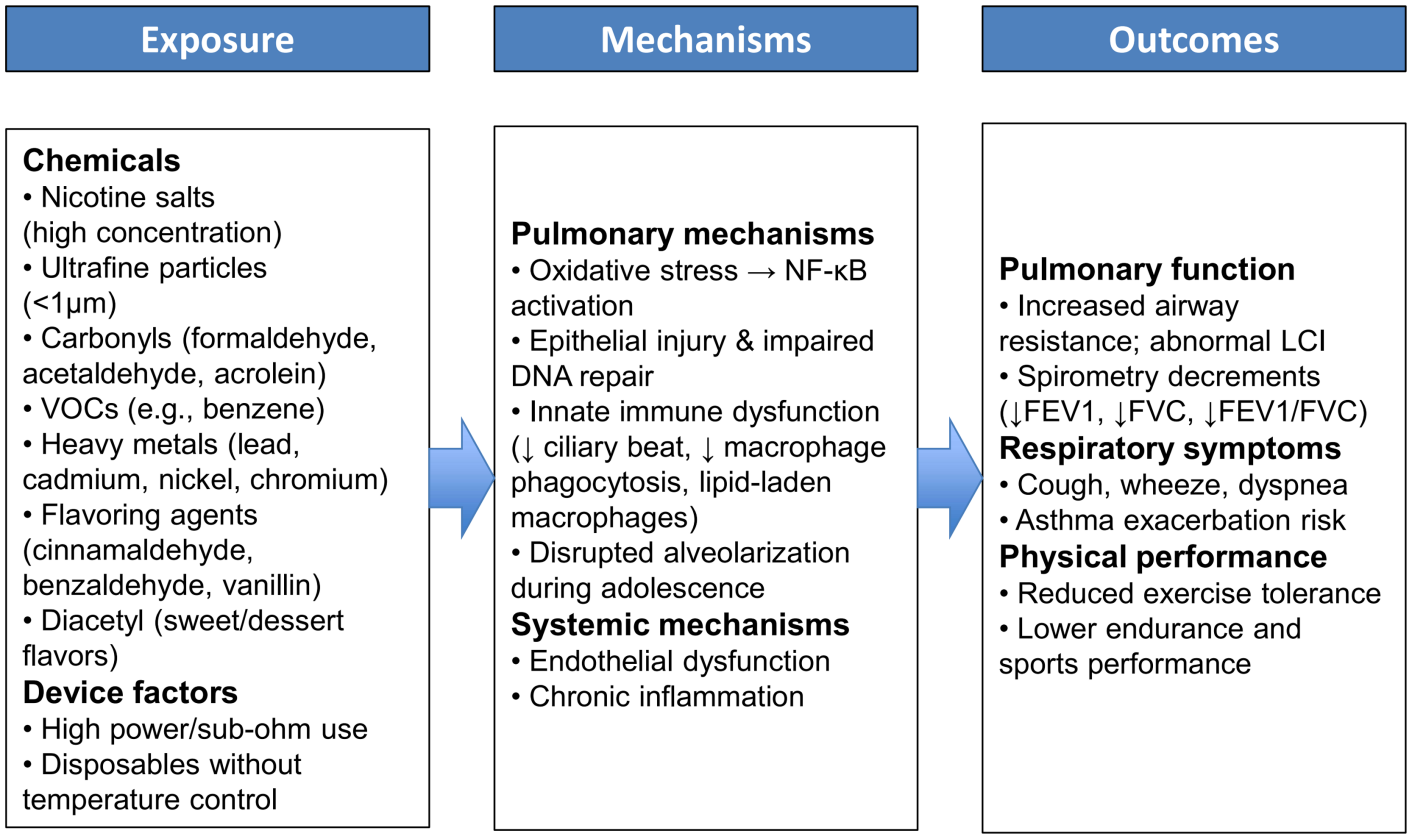
Conceptual framework illustrating the pathways linking adolescent E-cigarette exposure to pulmonary and functional outcomes.

While normal adolescent development supports the attainment of robust lung function and exercise capacity, the emergence of e-cigarette use introduces a new barrier to this trajectory. The interplay between exposure to e-cigarette toxicants, underlying biological mechanisms, and functional consequences is illustrated in Figure 2. This framework underscores how adolescent vaping can progress from chemical exposures to pulmonary injury and impaired physical performance. E-cigarette aerosol contains numerous potentially harmful substances that can be inhaled deep into young lungs. Inhalation of these aerosols can irritate and injure the delicate respiratory tissues of adolescents, whose lungs are still maturing. Indeed, the Centers for Disease Control and Prevention cautions that if teens vape, they breathe in tiny particles that can harm their lungs and damage lung function over time (71). There is growing evidence that habitual use of e-cigarettes interferes with normal lung growth and function in this age group.

**Figure 3:**
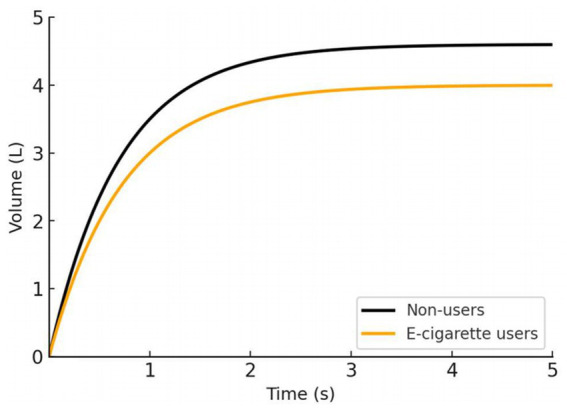
Comparison of spirometry time-volume curves between young adult non-users and e-cigarette users, adapted from reference 76. E-cigarette users demonstrated significantly lower ${\mathrm{FEV}}_{1}(n=65,3.02\pm 0.50\;L,p<0.001)$ and FVC(4.00±0.47L,p<0.001) than non-smokers $\left(n=63,;{\mathrm{FEV}}_{1}:3.51\pm 0.57\;L,;\mathrm{FVC}:4.57\pm 0.50\;L\right)$ .

Controlled laboratory studies of acute e-cigarette exposure have noted some immediate physiological effects on the airways of young people. Shortly after vaping, research has documented increased airway resistance and inflammation in the respiratory tract, as well as reports of transient breathing difficulty (72–74). By contrast, standard spirometric indices (FEV₁, FVC) may not show an instant drop after one vaping session in healthy individuals (75). This suggests that early e-cigarette effects might be subtle or primarily affect small airway function and respiratory comfort rather than gross lung volumes in the short term. However, when exposure is repeated regularly, even modest impairments could accumulate and become measurable. Emerging observational data indicate that adolescents and young adults who use e-cigarettes regularly tend to have lower lung function levels than those who never vape. For example, a cross-sectional study of young people found that e-cigarette users had significantly reduced spirometric lung volumes: average FEV₁ was only about 3.0 liters in vapers versus 3.5 liters in non-smokers, and mean FVC was 4.0 L in vapers versus 4.6 L in non-users (76) (Figure 3). Notably, the e-cigarette users in this study also showed a lower FEV₁/FVC ratio (75% vs. 79%), which is an early sign of airflow obstruction in the airways. Such findings suggest that regular vaping could be causing mild chronic airway narrowing or injury, even in otherwise healthy young adults. Studies have reported that youth who use e-cigarettes are more likely to experience recurrent asthma exacerbations and respiratory issues, implying that vaping may trigger or exacerbate airway disease in vulnerable adolescents (77). Over the long term, any vaping-induced decrement in attained peak lung function is concerning, because lower maximized FEV₁ or FVC in early adulthood is associated with higher risks of respiratory and cardiovascular complications later in life (78).

In summary, while research is still ongoing, current evidence points to e-cigarette use having a detrimental impact on pulmonary function development in youth. Regular vaping is biologically plausible to stunt the normal growth in lung capacity and to introduce chronic inflammatory changes in airways, thereby undermining the pulmonary foundation that is vital for both health and athletic endeavors in young people.

### 6.3 Respiratory symptoms and exercise performance in youth who vape

Beyond objective lung function measurements, e-cigarette use in adolescents has been associated with a range of respiratory symptoms that can directly impede physical activity and exercise performance. Multiple studies have reported that young e-cigarette users are more likely to experience symptoms such as wheezing, chest tightness, coughing, and shortness of breath (79, 80). In a recent longitudinal study of US high school students, those who had vaped in the past month had 81% higher odds of wheezing, roughly double the odds of bronchitis-like chronic cough, and 78% higher odds of experiencing shortness of breath compared to never-users (80). Similarly, an analysis of young adult participants (ages 18–24) in the Population Assessment of Tobacco and Health (PATH) study found that current e-cigarette users were significantly more likely to report respiratory issues including wheezing in the chest, difficulty breathing during exercise, and nightly cough, relative to peers who did not use e-cigarettes (81). Notably, even former e-cigarette users (those who quit) showed a higher prevalence of these symptoms than never-users, although those who continued vaping had the highest symptom odds, indicating a possible dose–response whereby ongoing exposure maintains or worsens respiratory symptoms (81). The presence of chronic shortness of breath and exercise-induced breathing difficulty in otherwise healthy teens is especially problematic, as it can limit their willingness or ability to participate in sports and vigorous activities. Previous research has shown that individuals who use e-cigarettes or smoke tobacco, particularly dual users, tend to exhibit lower levels of physical fitness. The respiratory irritation from inhaling vapes chemicals (for example, propylene glycol and flavoring agents known to cause airway irritation) likely underlies these subjective symptoms. Over time, this can translate into less engagement in exercise and deconditioning, creating a negative cycle of poor respiratory fitness.

Importantly, beyond self-reported symptoms, objective tests of exercise performance are beginning to reveal deficits in young e-cigarette users. In a large cohort of active young men, dual users demonstrated significantly poorer performance in cardiorespiratory endurance and muscular fitness tests compared to never users, including slower 2-mile run times and fewer push-ups and sit-ups performed, even after adjusting for physical training habits (82). Slower run times and reduced muscular endurance in the vaping group underscore that the effects of e-cigarettes extend beyond the lungs to impact whole-body fitness. Possible mechanisms include vaping-induced lung function limitations reducing oxygen supply during exertion, as well as systemic effects of vaping (such as carbon monoxide exposure, blood vessel dysfunction, or chronic inflammation) hampering exercise capacity (83). Nicotine itself can raise resting heart rate and blood pressure, which may reduce exercise efficiency and recovery in young users (84). The cumulative impact of these issues is that adolescents who vape may find it harder to engage and excel in physical activities.

In summary, the evidence to date indicates that e-cigarette use by adolescents can provoke respiratory symptoms and measurable decrements in exercise performance, thereby linking vaping to both physiological and functional harms. From chronic wheezing and shortness of breath to reduced aerobic capacity and endurance, vaping poses a threat to the pulmonary health and physical activity levels of youth. By compromising the very lung function that adolescents are in the process of maximizing, e-cigarette use may diminish their ability to lead active, healthy lives. Consequently, public health authorities and clinicians strongly advise that adolescents avoid e-cigarettes in order to safeguard their developing lungs and maintain their capacity for exercise and sport. Each prevented vaping habit in a teen may help preserve their pulmonary function and keep them on track for a healthier, more active adulthood free of unnecessary breathlessness and limitations.

## 7 Limitations and gaps

Many current studies on adolescent e-cigarette use, pulmonary function, and physical activity have significant methodological limitations. Research to date is dominated by cross-sectional designs and relies heavily on self-reported data for both e-cigarette exposure and health outcomes. This approach lacks a temporal framework to establish causality and is prone to reporting biases. Few studies incorporate objective clinical measures of lung function or fitness, leading to concerns about inconsistent or non-standardized assessment across studies. As a result, the true impact of adolescent vaping on pulmonary physiology may be under- or over-estimated due to these design constraints.

A related concern is the shortage of long-term and longitudinal data. E-cigarettes are a relatively recent phenomenon, and there have been virtually no long-term cohort studies following adolescent vapers into adulthood. Most available data reflect short-term observations, making it difficult to evaluate chronic effects on lung development or endurance capacity. Without prospective longitudinal research, questions remain about whether early e-cigarette use leads to lasting decrements in spirometry indices or exercise performance later in life. The literature’s emphasis on short study horizons means we do not yet know if any subclinical pulmonary changes in youth might progress to clinically significant deficits over time. Longitudinal studies with extended follow-up are critically needed to clarify these long-term trajectories.

Another major gap is the lack of mechanistic studies in adolescent populations. Ethical and practical barriers limit invasive physiological research in minors, so most mechanistic insights into e-cigarette effects come from adult or animal studies. Consequently, little is known about how vaping affects the developing lungs and airways at a cellular or molecular level in youths. Key questions regarding inflammatory responses, airway reactivity, or lung growth in adolescent vapers remain largely unanswered. The effects of e-cigarette use on objective cardiopulmonary endpoints in teenagers remain essentially unstudied. This gap hampers our understanding of cause-and-effect pathways, as we must extrapolate from adult data that may not fully generalize to younger, still-developing respiratory systems.

Finally, there is very limited evidence linking adolescent e-cigarette use with objectively measured exercise performance or physical activity levels. To date, most research on vaping and physical health in youth has focused on self-reported symptoms or general health indicators, rather than direct fitness assessments. Studies have only just begun to explore whether vaping translates into diminished exercise capacity in young people. Few studies have quantified metrics like aerobic endurance, pulmonary exercise testing, or daily activity via wearables in adolescent vapers. Because of this scarcity of adolescent-specific research, some of the available findings on physical performance are derived from young-adult or mixed-age populations (typically aged 18–25 years). While these data provide useful physiological context, their applicability to adolescents should be interpreted with caution due to developmental differences in lung maturation and exercise capacity. This lack of evidence leaves it uncertain whether vaping adolescents experience measurable decrements in sports performance or activity levels compared to their peers.

In summary, these limitations, predominantly cross-sectional and self-reported study designs, absence of long-term longitudinal research, minimal mechanistic investigation in youths, and sparse data on exercise outcomes, constrain our current understanding of how e-cigarette use may affect adolescent pulmonary function and physical activity. Addressing these gaps through rigorous future studies (e.g., well-controlled longitudinal cohorts, clinical trials, and mechanistic experiments adapted for adolescent participants) will be essential to draw more definitive conclusions and inform evidence-based public health guidance for youth vaping.

## 8 Conclusion

Current evidence suggests that adolescent e-cigarette use is associated with adverse effects on respiratory health, including increased respiratory symptoms, reduced lung function, and early signs of diminished exercise capacity. These findings are biologically plausible given the toxicants in e-cigarette aerosol and the vulnerability of developing lungs. However, the literature remains limited: most studies are cross-sectional, rely on self-report, and provide little longitudinal data to confirm causality or long-term outcomes.

Future research should prioritize prospective cohort studies and objective measurements of pulmonary function and exercise capacity, while mechanistic investigations in adolescent populations are needed to clarify causal pathways. Importantly, standardized protocols for assessing both respiratory outcomes and physical performance should be adopted to reduce heterogeneity across studies.

In parallel with research, urgent public health strategies—such as stricter regulation of flavored products, youth-targeted prevention campaigns, and clinical screening for vaping-related symptoms—are required to mitigate the risks. Protecting adolescents during this critical developmental stage is essential to ensure they reach their full pulmonary potential and maintain the capacity for an active and healthy life.
